# β-defensin 118 attenuates inflammation and injury of intestinal epithelial cells upon enterotoxigenic *Escherichia coli* challenge

**DOI:** 10.1186/s12917-022-03242-3

**Published:** 2022-04-19

**Authors:** Qingqing Fu, Qian Lin, Daiwen Chen, Bing Yu, Yuheng Luo, Ping Zheng, Xiangbing Mao, Zhiqing Huang, Jie Yu, Junqiu Luo, Hui Yan, Jun He

**Affiliations:** 1 Institute of Animal Nutrition, Sichuan Agricultural University, Chengdu, Sichuan Province 611130 P. R. China; 2 Key Laboratory of Animal Disease-resistant Nutrition, Chengdu, Sichuan Province 611130 P. R. China

**Keywords:** Antimicrobial peptide, ETEC, inflammation, IPEC-J2 cells, NF-κB

## Abstract

**Background:**

Antimicrobial peptides including various defensins have been attracting considerable research interest worldwide, as they have potential to substitute for antibiotics. Moreover, AMPs also have immunomodulatory activity. In this study, we explored the role and its potential mechanisms of β-defensin 118 (DEFB118) in alleviating inflammation and injury of IPEC-J2 cells (porcine jejunum epithelial cell line) upon the enterotoxigenic *Escherichia coli* (ETEC) challenge.

**Results:**

The porcine jejunum epithelial cell line (IPEC-J2) pretreated with or without DEFB118 (25 μg/mL) were challenged by ETEC (1×10^6^ CFU) or culture medium. We showed that DEFB118 pretreatment significantly increased the cell viability (*P*<0.05) and decreased the expressions of inflammatory cytokines such as the interleukin-1β (IL-1β), interleukin-6 (IL-6), and tumor necrosis factor-α (TNF-α) in IPEC-J2 cells exposure to ETEC (*P*<0.05). Interestingly, DEFB118 pretreatment significantly elevated the abundance of the major tight-junction protein zonula occludens-1 (ZO-1), but decreased the number of apoptotic cells upon ETEC challenge (*P*<0.05). The expression of caspase 3, caspase 8, and caspase 9 were downregulated by DEFB118 in the IPEC-J2 cells exposure to ETEC (*P*<0.05). Importantly, DEFB118 suppressed two critical inflammation-associated signaling proteins, nuclear factor-kappa-B inhibitor alpha (I*κ*B-α) and nuclear factor-kappaB (NF-*κ*B) in the ETEC-challenged IPEC-J2 cells.

**Conclusions:**

DEFB118 can alleviate ETEC-induced inflammation in IPEC-J2 cells through inhibition of the NF-κB signaling pathway, resulting in reduced secretion of inflammatory cytokines and decreased cell apoptosis. Therefore, DEFB118 can act as a novel anti-inflammatory agent.

## Introduction

The intestinal epithelium not only acts as the major site for nutrient absorption but also acts as the primary physical barrier against a wide variety of endogenous and exogenous harmful substances in the gastrointestine [1–3]. Disruption of the intestinal epithelium by various bacterial pathogens may result in inflammation and severe diarrhea in neonatal animals [4, 5]. For instance, the enterotoxigenic *Escherichia coli* (ETEC) has been identified as the most critical bacterial causing post-weaning diarrhea (PWD) [6–8]. Colonization and proliferation of ETEC strains in the intestine produce a large number of enterotoxins that act on the small intestine and lead to the secretion of fluids and electrolytes, causing diarrhea [9, 10]. In last decades, antibiotics have been widely used to prevent PWD. However, long-term or overdose utilization of antibiotics may lead to the developing of drug resistance [11–13]. Therefore, novel avenues to prevent various bacteria-induced inflammation and intestinal epithelium disruption are urgently needed.

Previous studies indicated that the intestinal epithelium can also serve as a vital immune organ, and the intestinal epithelial cells can secrete a variety of bioactive substances (e.g. antimicrobial peptides) that play important roles in regulating immunity and intestinal health [14–17]. Defensins are diverse members of a large family of antimicrobial peptides, contributing to the antimicrobial action of granulocytes, mucosal host defence in the small intestine and epithelial host defence in the skin and elsewhere [18]. Previous studies indicated that defensins can be divided into α, β, and θ subclasses according to their disulfide bonding, genomic organization, and tissue distribution [19–22]. Amongst the three types of defensins, the β-defensins have been the most extensively studied to date. β-defensins are usually translated from characteristic two exon gene structures, the first of which encodes a prepropeptide while the mature peptide is encoded by the second exon, containing the six-cysteine motif [23]. Importantly, β-defensins were traditionally viewed as exclusively antimicrobial molecules, as their induction in response to diverse bacterial, viral, parasitic, and fungal infections was widely reported [24–26]. There are also reports showing that β- defensins can also inhibit inflammation. For instance, β-defensin 129 was reported to attenuate intestinal inflammation and epithelial atrophy in rat exposure to bacterial endotoxin [27]. Human β-defensin 114 regulates lipopolysaccharide (LPS)-mediated inflammation and protects sperm from motility loss [28].

β-defensin 118(DEFB118) is a novel antimicrobial peptide that can obtain from caput and efferent ducts of epididymis [29]. Interestingly, DEFB118 can disrupt the membrane of *E. coli* and change their morphology of the bacterial surface [30]. Moreover, our previous study found that DEFB118 exhibited antimicrobial activity against both Gram-negative and Gram-positive bacteria [31]. Although DEFB118 has shown antimicrobial activity, the exact role of DEFB118 in regulating mucosal immunity and intestinal health are unknown. In the present study, we explored the role of DEFB118 in alleviating inflammation and injury of intestinal epithelial cells during exposure to ETEC. The mechanisms behind the DEFB118 regulated actions have also been partially investigated.

## Materials and Methods

### Strains and Vectors

The *E. coli* DH5α and *E. coli* Orgami B (DE3) strains were purchased from TIANGEN (Beijing, China). The pET32a (+) was purchased from Merck KGaA (Darmstadt, Germany). ETEC (O149: K91, K88ac) was purchased from China Veterinary Culture Collection Center (Beijing, China).

### Plasmid Construction, Expression, and Purification of DEFB118

The target gene DEFB118 was synthesized and introduced *Eco* RΙ and *Not* Ι restriction sites at the 5'and 3'ends of the target gene by Tsingke Biological Technology Co., Ltd. (Chengdu, China). The DEFB118 fragment was cloned into the expression vector pET32a (+) after double enzymatic digestion by *Eco* RI and *Not* I (Japanese Takara). The resulting plasmid pET32a(+)-DEFB118 was transformed into *E. coli* Orgami B (DE3) and induced by 1.0 mM isopropyl β-d-1-thiogalactoside (IPTG). After incubation for 4 h at 28°C, the bacteria were collected by centrifugation at 8000×g for 20 min at 4°C and lysed by lysis buffer [500 mM NaCl, 20 mM Tris, 0.1% Triton X-100, 1 mM PMSF, Lysozyme 0.2 mg/mL, 10 U/mL DNase (pH 7.5)] for 30 min at 4°C. Then, schizolytic cells were sonicated (4 s pulse and 8 s interval; 30 cycles; Sonics-Vibra cell, USA). The supernatant of the cell lysate resulting from centrifugation at 15000 × g for 30 min was applied to a Ni^2+^-NTA column (Sangon Biotech, Shanghai). After washing to baseline absorbance with Binding buffer (20 mM Tris-HCI, 8 M urea, 0.5 M NaCl, 5 mM imidazole, pH 8.0), the column was washed with Elution Buffer (20 mM Tris-HCI, 8 M urea, 0.5 M NaCl, 500 mM imidazole, pH 8.0) at a flow rate of 1 mL/min. The fractions were collected and dialyzed with sterile saline solution (0.09% [wt/vol] NaCl in distilled water). The purified DEFB118 was run on 12% SDS-PAGE. The rest was stored at −80°C after quantified with the BCA assay (Beyotime, China).

### Cell culture and treatment

The porcine jejunum epithelial cell line (IPEC-J2) was obtained from the American Type Culture Collection (ATCC, Manassas, VA, USA). The cells were cultured in DMEM F12 medium supplemented with 10% FBS (Fetal bovine serum), 100 U/mL penicillin, and 100 μg/mL streptomycin at 37°C with 5% CO_2_ in a humidified atmosphere. In addition, the cells were seeded in plates once every 2–3 days to achieve 80% confluence. After incubated with antimicrobial peptide DEFB118 (25 μg/mL) for 12 h or BAY11-7082 (an inhibitor of IκB-α phosphorylation and NF-κB) for 2 h, then cells were challenged with 1×10^6^ CFU/well ETEC for 1 h or 2.5 h (only for assessment of apoptosis), It is worth noting that when challenged with ETEC, the cells were cultured in DMEM F12 medium supplemented with 2% FBS (without any antibiotics).

### Cell viability assay

MTT (Sigma, USA) was used to evaluate cell viability. Briefly, IPEC-J2 cells seeded in 96-well plates (Corning, USA) were incubated with 20 μL MTT for 4 h immediately after treatment. Next, the culture medium containing MTT was aspirated, 150 μL DMSO was added and oscillated at low speed for 10 min. Last, the optical density (OD) of the wells was read at 570 nm by a microplate reader (SpectraMax 190, Molecular Devices, California, USA). Cell viability (%) = (OD_treatment group_ - OD_blank group_)/(OD_control group_ - OD_blank group_) × 100.

### RNA extraction and RT-PCR

The total RNA was extracted from the IPEC-J2 cells by RNAiso Plus (Takara, Dalian, China) according to the manufacturer’s instructions. RNA concentration and purity were determined by the NanoDrop 2000 spectrophotometer at 260 and 280 nm (Thermo Fisher Scientific Inc., Waltham, MA, USA). And then cDNA was synthesized by a Reverse Transcriptase kit (Takara, Dalian, China). Quantitative PCR was performed by QuanStudio 6 Flex Real-Time PCR detection system (Applied Biosystems, Foster City, CA, USA) with a total of 10 μL of assay solution containing 5 μL SYBR Green mix (Takara), 0.2 μL Rox, 3 μL deionized H2O, 1 μL cDNA template, and 0.4 μL each of forward and reverse primers (Sangon, China). The relative gene expressions compared with the housekeeping gene β-actin were calculated by 2^- ∆∆ct^ [32]. The primer sequences show in Table 1.

**Table 1:** Primer sequences for quantitative real-time polymerase chain reaction

Gene^a^	Primer sequence (5′-3′)	Accession Number.
*IL-1β*	Forward: AAAGCCCAATTCAGGGACCCTAC Reverse: CCATCACTTCCTTGGCGGGTT	NM_214055.1
*IL-6*	Forward: AGGGAAATGTCGAGGCTGTGC Reverse: CCGGCATTTGTGGTGGGGTT	NM_214399.1
*TNF-α*	Forward: TTCGAGGTTATCGGCCCCCA Reverse: GTGGGCGACGGGCTTATCTG	NM_214022.1
*CASP3*	Forward: GGAATGGCATGTCGATCTGGT Reverse: ACTGTCCGTCTCAATCCCAC	NM_214131.1
*CASP8*	Forward: TCTGCGGACTGGATGTGATT Reverse: TCTGAGGTTGCTGGTCACAC	NM_001031779.2
*CASP9*	Forward: AATGCCGATTTGGCTTACGT Reverse: CATTTGCTTGGCAGTCAGGTT	XM_003127618.4
*β-actin*	Forward: TGGAACGGTGAAGGTGACAGC Reverse: GCTTTTGGGAAGGCAGGGACT	XM_003124280.5

^**a**^ *IL-1β* interleukin-1β, *IL-6* interleukin-6, *TNF-α* tumour necrosis factor-α, *CASP3* caspase 3*, CASP8* caspase 8*, CASP9 caspase 9, β-actin,* beta-actin

### Assessment of apoptosis by flow cytometry

Apoptotic IPEC-J2 cells were detected by an Annexin V-PE/7-AAD Apoptosis Detection Kit (B&D Pharmingen, USA) or an Annexin V-FITC/PI Apoptosis Detection Kit (BD Biosciences, USA) according to the manufacturer's instructions. Cells seeded in 12-well plates (Corning, USA) were harvested by 0.25% trypsin without EDTA after the treatment. After centrifuged at 350 ×g for 10 min, the cells were washed with ice-cold PBS, centrifuged again, and resuspended with 100 μL 1× binding buffer. Next, 2 μL PE Annexin V or AnnexinV-FITC and 2 μL 7-ADD or PI were added into the cells and they were incubated for 15 min at room temperature in the dark. Finally, 400 μL 1× Binding Buffer was added to the mixture and cell apoptosis was evaluated by CytoFlex flow cytometer (Beckman Coulter, Inc., Brea, CA, USA).

### Immunofluorescence

IPEC-J2 cells were seeded on coverslips treated with concentrated sulfuric acid placed in 12-well cell culture plates at a density of 2 × 10^5^ cells/well and cultured to 80–90% confluence. Subsequently, the cells were treated with reagents (DEFB118 and BAY11-7082) and ETEC according to the experimental design. After washed with ice-cold PBS, cells were fixed with 4% paraformaldehyde 15 min at room temperature. After washed three times with PBS (pH 7.4) for 2 min each time, cells incubated overnight with the primary antibody at 4°C in the dark (rabbit anti-ZO-1; Novus; Cat no.: NBP1-85047; 1:200). Next, cells were washed three times with PBS (pH 7.4) for 2 min each time and incubated for 2 h at room temperature with the appropriate secondary antibody (Alexa Fluor 488 conjugated goat anti-rabbit immunoglobulin; CST; Cat no.:4412S; 1:1000). Finally, the cells were washed three times with PBS (pH 7.4) for 2 min each time and counterstained with DAPI (Sigma-Aldrich). cells were imaged using a confocal scanning microscope (NIKON ECLIPSE TI-SR)

### Total protein extraction and western blot analysis

The total protein was extracted from the IPEC-J2 cells by cell lysis buffer for western blot (WB) analysis. After the protein concentration was determined by a BCA assay kit (Beyotime Institute of Biotechnology, Shanghai, China), the supernatants were diluted with 4 × Laemmli sample buffer (Bio-Rad Laboratories, Inc., Hercules, CA, USA) containing 10% β-mercaptoethanol and denatured at 98°C for 10 min. Then, equal amounts of proteins in boiled samples were separated via 10% sodium dodecyl sulfate-polyacrylamide gel electrophoresis (SDS-PAGE) and transferred onto 0.45 μm polyvinylidene fluoride (PVDF) membranes (Merck Millipore Ltd., Tullagreen, Ireland). Next, the PVDF membranes were blocked with 5% non-fat dry milk at room temperature for 1 h. After being washed in TBS/T three times for 10 min each, the membranes were incubated with specific primary antibodies [ZO-1 (Novus; Cat no.: NBP1-85047; 1:1000), p-NF-κB p65 (CST; Cat no.:3033S; 1:1000), NF-κB p65 (CST; Cat no.:6956S; 1:1000), IκBα (CST; Cat no.:4814S; 1:1000), p-IκBα (Invitrogen; Cat no.:MA5-15224; 1:1000), GAPDH (CST; Cat no.:2118S; 1:1000)] at 4°C for over-night under gentle agitation. After being washed in TBS/T three times for 10 min each, the membranes were incubated with the corresponding secondary antibodies [anti-rabbit IgG (CST; Cat no.7074S; 1:2500), anti-mouse IgG (CST; Cat no.7076S; 1:2500)] for 1 h at room temperature. Finally, after washing thrice with TBS/T, the PVDF membranes were treated with Clarity™ Western ECL Substrate (Bio-Rad Laboratories, Inc.). The protein bands were photographed by the ChemiDocTMXRS+ Imager System (Bio-Rad Laboratories, Inc.). The intensity of the protein bands was quantified with Quantity One software (Bio-Rad Laboratories, Inc.), and the results were expressed as the abundance of the target protein relative to that of the reference protein (GAPDH).

### Statistics analysis

All statistical analysis was performed using SPSS26.0 software. Data were expressed as the mean ± standard error (SEM). Statistical analysis was carried out using Two-way analysis of variance (ANOVA) followed by LSD multiple comparison test. *P*<0.05 was considered statistically significant. Image production using GraphPad Prism software (Version 8. GraphPad Software Inc., CA, USA).

## Results

### Expression and purification of DEFB118

**Fig. 1.**
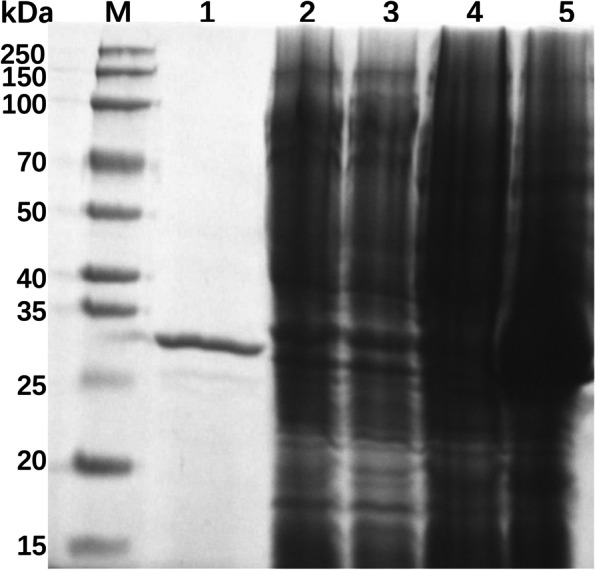
SDS-PAGE analysis of DEFB118 produced by *E. coli* Rosetta. M, 250 kDa protein markers. 1, purification of DEFB118; 2, *E. coli* Origami B (DE3)-pET32a(+) did not induce by 1 mmol/L IPTG for 4 h at 28°C. 3, *E. coli* Origami B (DE3)-pET32a(+) induced by 1 mmol/L IPTG for 4 h at 28°C. 4, *E. coli* Origami B (DE3)-pET32a(+)- DEFB118 did not induce for 6 4 at 28°C; 5, *E. coli* Origami B (DE3)- pET32a(+)-DEFB118 induced by 1 mmol/L IPTG for 4 h at 28°C

As shown in Fig. 1, the cell extracts from *E. coli* Origami B (DE3) harboring the plasmid pET32a (+)-DEFB118 showed a clear band with molecular weight about 30 kDa. No bands were observed in the extract from the un-induced control strain or *E. coli* harboring the empty plasmids. The molecular weight of DEFB118 is consistent with the predicted size. The crude recombinant proteins were extracted from *E. coli* and then purified by Ni^2+^-NTA affinity chromatography. The result from SDS-PAGE verified successful purification, as only one clear band with molecular weight about 30 kDa was observed (Fig. 1).

### Influences of ETEC challenge on the viability and inflammatory response of IPEC-J2 cells

**Fig. 2.**
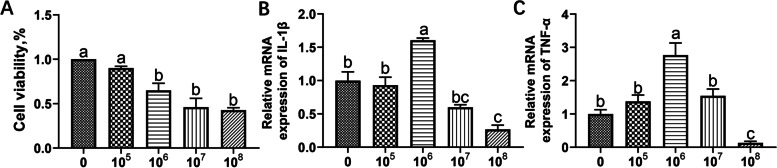
Influences of ETEC challenge on viability and inflammatory response of IPEC-J2 cells. The IPEC-J2 cells were treated with ETEC at different concentrations (0, 10^5^, 10^6^, 10^7^, 10^8^ CFU/well) for 1 h or 3 h. **A** Cell viability; **B** the expression of IL-1β; **C** the expression of TNF-α. *n*=3. Data were presented as mean ± standard error (SEM). ^a-c^ Values within a column differ if they do not share a common superscript (*P*<0.05)

To explore the influence of ETEC challenge on cell viability, the IPEC-J2 cells were treated with ETEC at different concentrations (0, 10^5^, 10^6^, 10^7^, 10^8^ CFU/well) for 3 h. As shown in Fig. 2A, the viability of the cells was significantly decreased upon ETEC challenge at a moderate or higher dose (10^6^, 10^7^, and 10^8^ CFU/well) (*P*<0.05). However, there were no significant differences in cell viability among the three groups (*P*>0.05). We also determined the influences of different doses of ETEC on the inflammatory response in the IPEC-J2 cells. As shown in Fig. 2B and C, the expression levels of inflammatory cytokines such as the IL-1β and TNF-α were significantly elevated in the cells upon ETEC challenge at a dose of 10^6^ CFU/well (*P*<0.05). As compared to this dose, a higher dose (10^7^ and 10^8^ CFU/well) significantly decreased their expression levels in the IPEC-J2 cells (*P*<0.05). Therefore, a moderate dose (10^6^ CFU/well) was used for further construction of challenge model.

### Effect of DEFB118 on cell viability and inflammatory responses of IPEC-J2 cells upon ETEC challenge

**Fig. 3.**
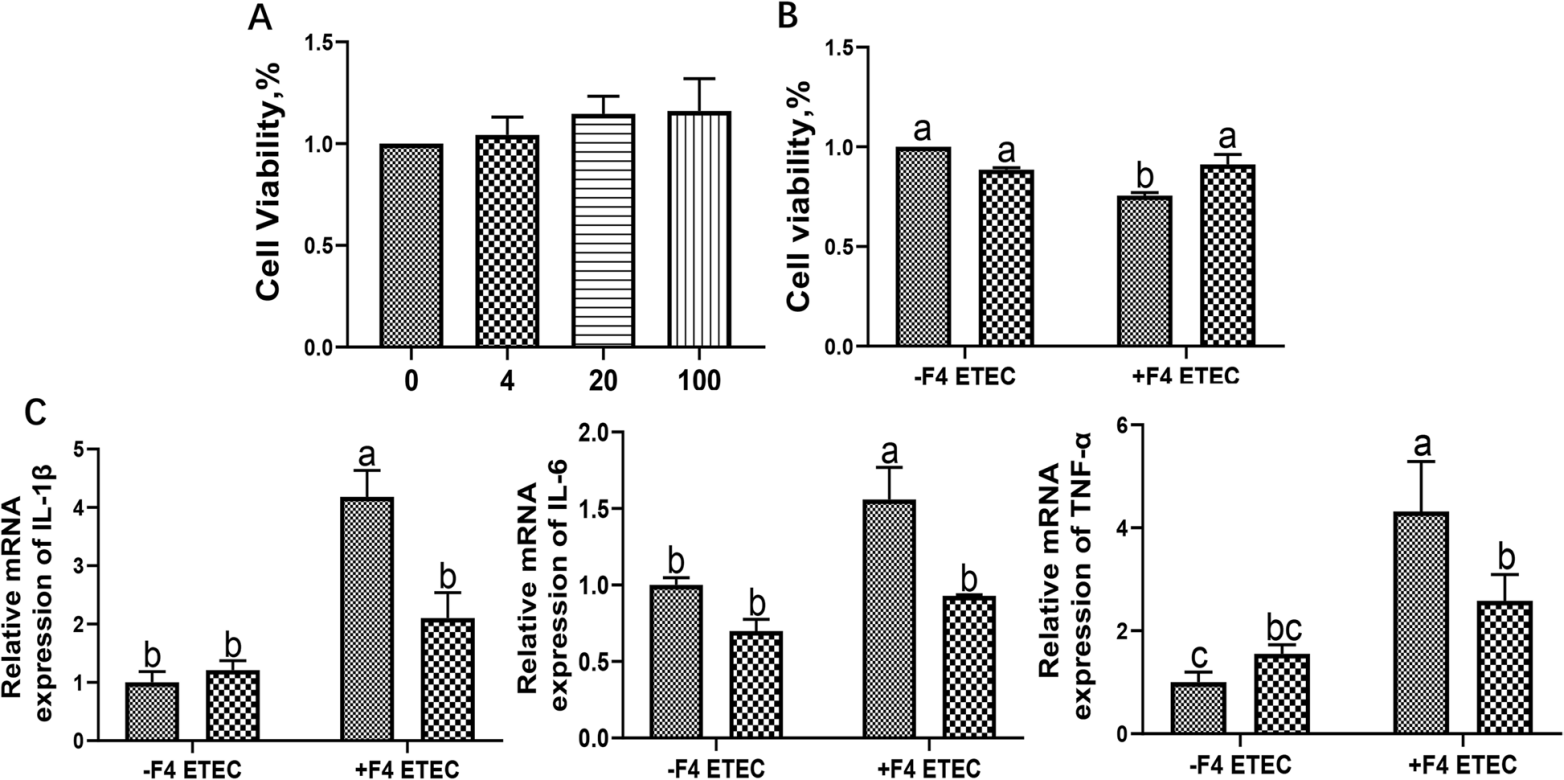
Effect of DEFB118 on cell viability and inflammatory responses of IPEC-J2 cells upon ETEC challenge. The IPEC-J2 cells were treated with DEFB118 at different concentrations (0, 4, 20, and 100 μg/mL) for 12 h. **A** Cell viability. The IPEC-J2 cells were treated with DEFB118 (25 μg/mL) for 12 h, followed by co-treatment with ETEC (1×10^6^ CFU) for 1 h. **B** Cell viability. **C** the expressions of IL-1β, IL-6 and TNF-α. *n*=3*.*Data were presented as mean ± standard error (SEM). ^a-c^ Values within a column differ if they do not share a common superscript (*P*<0.05). “ CON ” stand for “ Control ” “ DEFB ” stand for “ DEFB118 ” “ ETEC ” stand for “ ETEC ” “ ETECD ” stand for “ ETEC+DEFB118 ”

To explore the influence of DEFB118 on cell viability, the IPEC-J2 cells were treated with DEFB118 at different concentrations (0, 4, 20, and 100 μg/mL) for 12 h. Results showed that treatment with the cells with DEFB118 ranging from 4 to 100 μg/mL had no negative influence (toxic effect) on cell viability (Fig. 3A). Therefore, a moderate dose 25 μg/mL was used for further studies. As shown in Fig. 3B, ETEC challenge decreased the viability of the IPEC-J2 cells; however, pretreatment of the cells with 25 μg/mL DEFB118 significantly increased the cell viability upon ETEC challenge (*P*<0.05). Moreover, ETEC challenge significantly elevated the expression levels of inflammatory cytokines such as the IL-1β, IL-6, and TNF-α in IPEC-J2 cells (*P*<0.05). However, DEFB118 pretreatment significantly downregulated their expressions in the IPEC-J2 cells upon ETEC challenge (Fig. 3C).

### Effect of DEFB118 on tight junction protein abundance in IPEC-J2 cells upon ETEC challenge

**Fig. 4.**
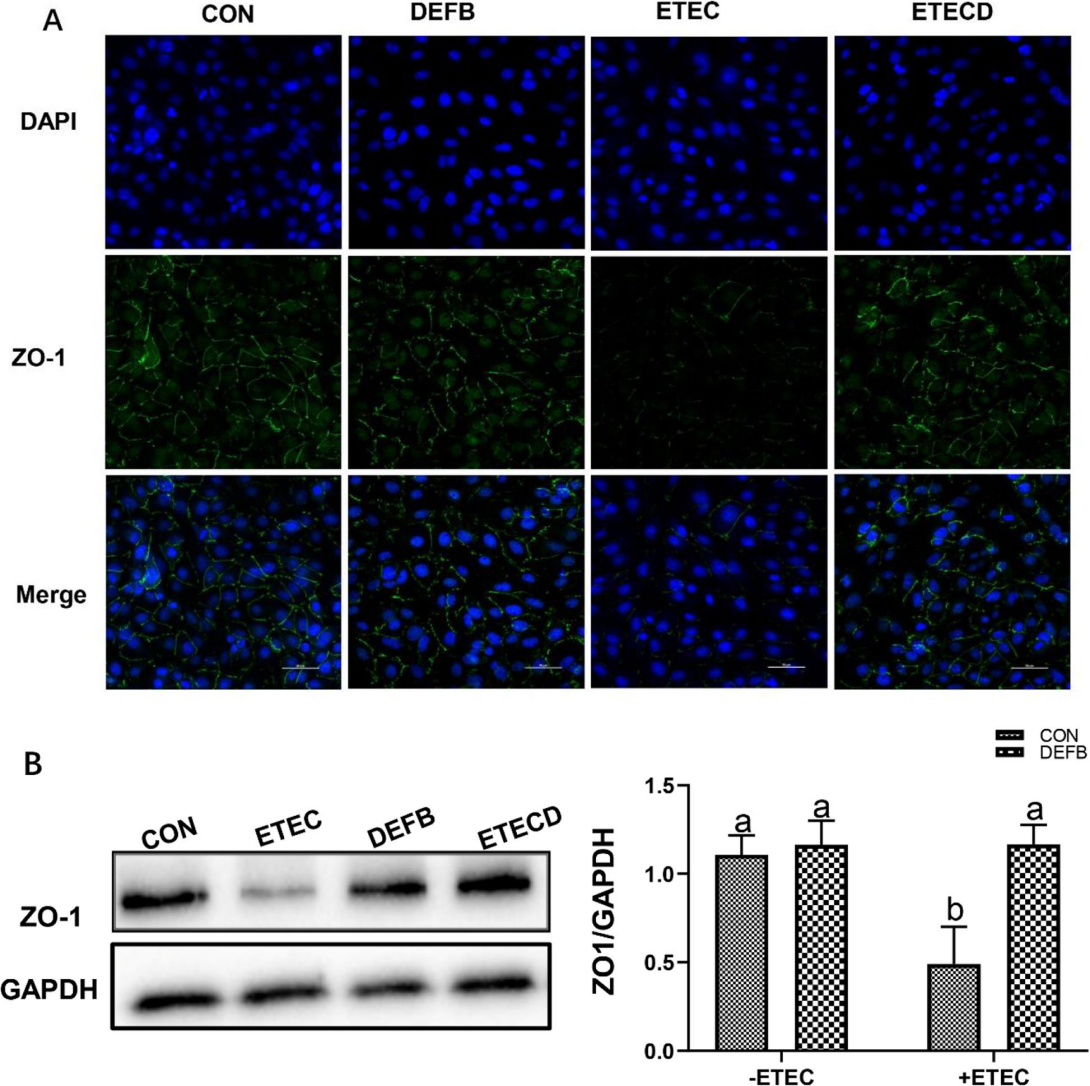
Effect of DEFB118 on tight junction protein distribution and abundance in IPEC-J2 cells upon ETEC challenge. IPEC-J2 cells pretreated with DEFB118 (25 μg/mL) for 12 h, followed by co-treatment with ETEC (1 × 10^6^ CFU) for 1 h. **A** Zonula occludens-1 (ZO-1) distribution in the IPEC-J2 cells (Immunofluorescence). **B** Western blot analysis of ZO-1 in the IPEC-J2 cells. Scale bar = 50 μm. *n*=3. Data were presented as mean ± standard error (SEM). ^a-b^ Values within a column differ if they do not share a common superscript (*P*<0.05). “ CON ” stand for “ Control ” “ DEFB ” stand for “ DEFB118 ” “ ETEC ” stand for “ ETEC ” “ ETECD ” stand for “ ETEC+DEFB118 ”

As shown in Fig. 4A, there was less staining of the major tight junction protein ZO-1 in the ETEC-challenged cells. However, the staining of the ZO-1 was enhanced by DEFB118 pretreatment in the ETEC-challenged cells. We also investigated the abundance of ZO-1 by using western blot assay. As shown in Fig. 4B, ETEC challenge decreased ZO-1 abundance in the IPEC-J2 cells; however, DEFB118 pretreatment significantly elevated its abundance in the ETEC-challenged cells (*P*<0.05).

### Effect of DEFB118 on apoptosis of IPEC-J2 cells upon ETEC challenge

**Fig. 5.**
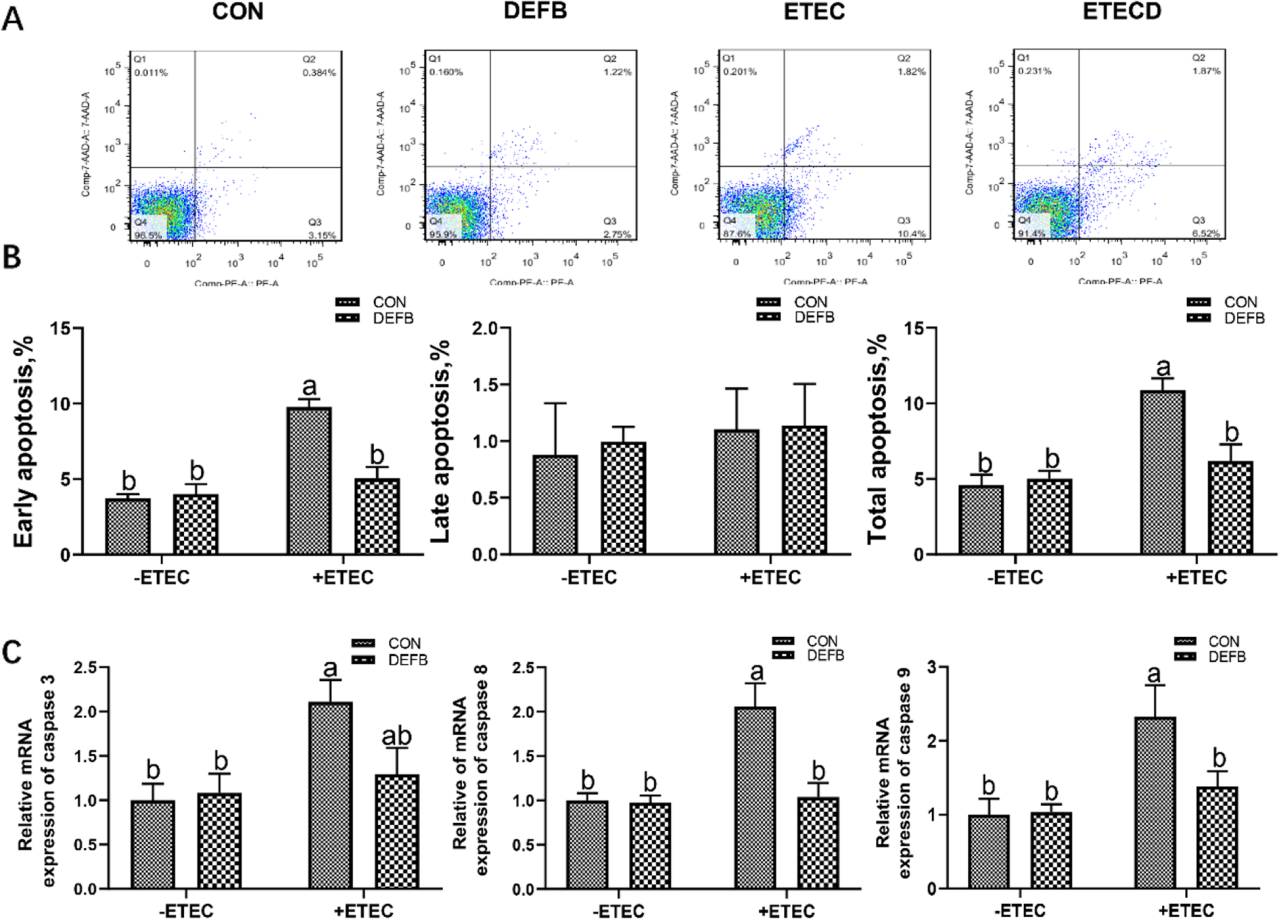
Effect of DEFB118 on apoptosis of IPEC-J2 cells upon ETEC challenge. IPEC-J2 cells pretreated with DEFB118 (25 μg/mL) for 12 h, followed by co-treatment with ETEC (1 × 10^6^ CFU) for 1 h or 2.5 h. **A** Flow cytometry analysis of apoptotic cells (Annexin V-PE/7-AAD). **B** Quantification of apoptotic cells from flow cytometry data. **C** the expressions of Caspase 3, Caspase 8 and Caspase 9. *n*=3. Data were presented as mean ± standard error (SEM). ^a-b^ Values within a column differ if they do not share a common superscript (*P*<0.05). “ CON ” stand for “ Control ” “ DEFB ” stand for “ DEFB118 ” “ ETEC ” stand for “ ETEC ” “ ETECD ” stand for “ ETEC+DEFB118 ”

As shown in Fig. 5A and B, ETEC challenge increased the early and total apoptosis rate in the IPEC-J2 cells; however, DEFB118 pretreatment significantly reduced the early and total apoptosis rate in the ETEC-challenged cells (*P*<0.05). ETEC challenge significantly elevated the expression levels of critical apoptosis-related genes such as caspase 3, caspase 8, and caspase 9 in the cells (Fig. 5C). However, both the expressions of caspase 8 and caspase 9 were significantly downregulated by DEFB118 in the ETEC-challenged cells (*P*<0.05).

### DEFB118 suppressed ETEC-induced cell apoptosis and inflammatory response via suppressing the IκB-α/NF-κB signaling

**Fig. 6.**
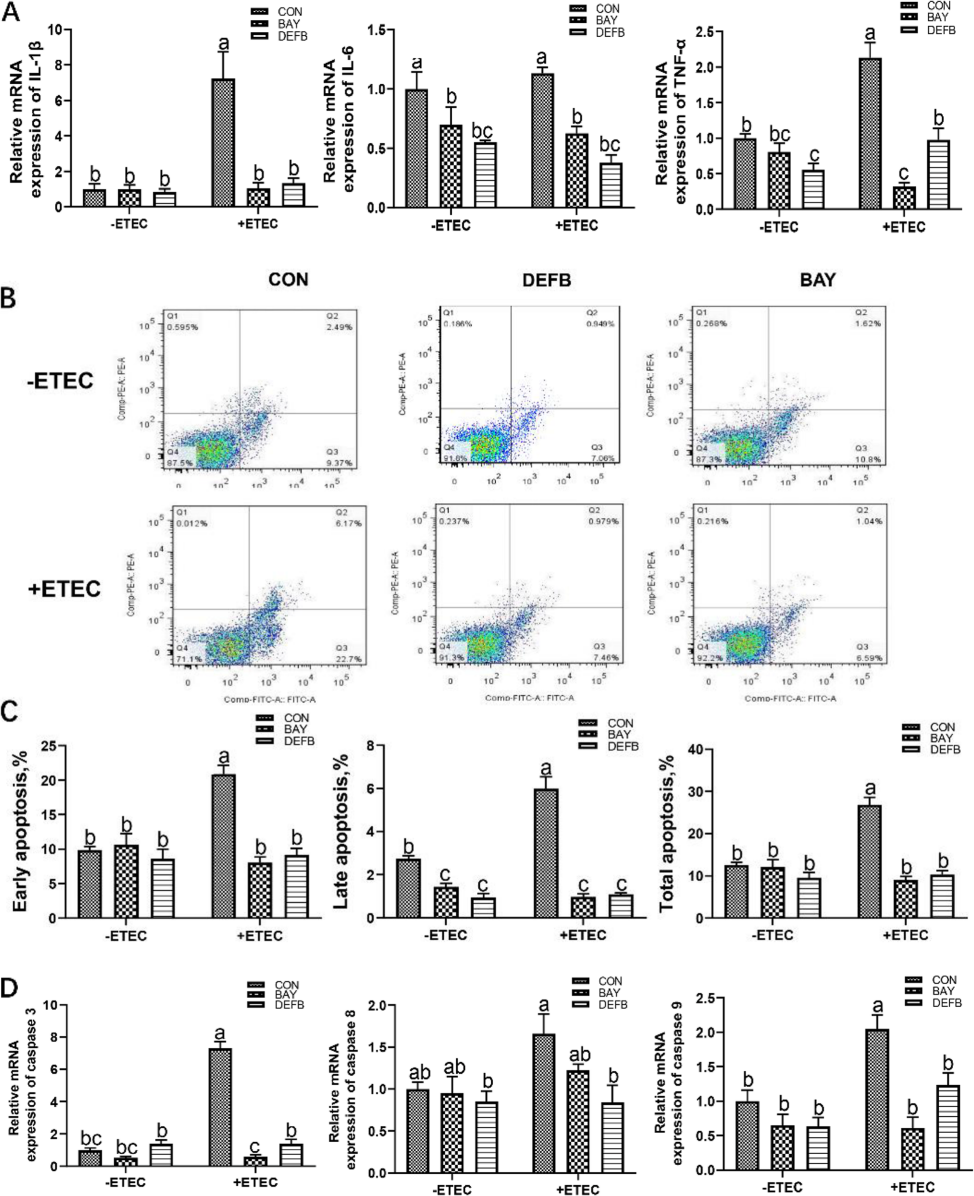
DEFB118 suppressed ETEC-induced cell apoptosis and inflammatory response via suppressing the IκB-α/NF-κB signaling. IPEC-J2 cells were pretreated with DEFB118 (25 μg/mL) for 12 h or with BAY11-7082 for 2 h , followed by co-treatment with ETEC (1 × 10^6^ CFU) for 1 h or 2.5 h. **A** the expressions of IL-1β, IL-6 and TNF-α. **B** Flow cytometry analysis of apoptotic cells (Annexin V-FITC/PI). **C** Quantification of apoptotic cells from flow cytometry data. **D** the expressions of Caspase 3, Caspase 8 and Caspase 9. *n*=3*.* Data were presented as mean ± standard error (SEM). ^a-c^ Values within a column differ if they do not share a common superscript (*P*<0.05). “ CON ” stand for “ Control ” “ BAY ” stand for “ BAY11-7082 ” “ DEFB ” stand for “ DEFB118 ” “ ETEC ” stand for “ ETEC ” “ ETECB ” stand for “ BAY11-7082+DEFB118 ” “ ETECD ” stand for “ ETEC+DEFB118 ”

**Fig. 7.**
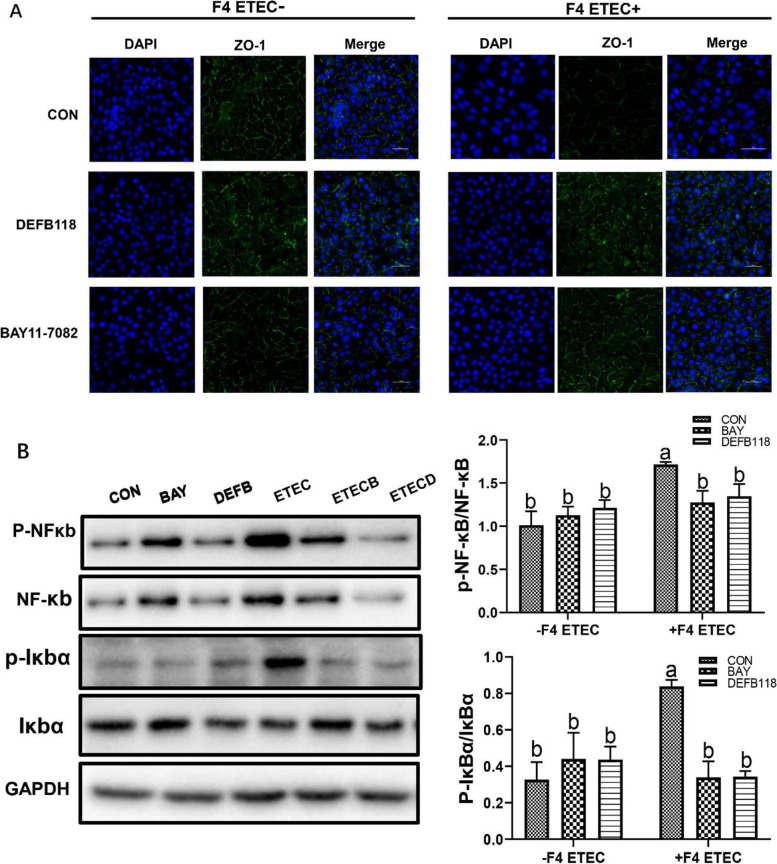
DEFB118 suppressed ETEC-induced the jury of IPEC-J2 and phosphorylation of IκB-α and NF-κB. IPEC-J2 cells were pretreated with DEFB118 (25 μg/mL) for 12 h or with BAY11-7082 for 2 h, followed by co-treatment with ETEC (1 × 10^6^ CFU) for 1 h. **A** Zonula occludens-1 (ZO-1) distribution in the IPEC-J2 cells (immunofluorescence). **B** Western blot and quantitative analysis of phosphorylation of IκBα and NF-κB. Scale bar = 50 μm. *n*=3. Data were presented as mean ± standard error (SEM). ^a-b^ Values within a column differ if they do not share a common superscript (*P*<0.05). “ CON ” stand for “ Control ” “ BAY ” stand for “ BAY11-7082 ” “ DEFB ” stand for “ DEFB118 ” “ ETEC ” stand for “ ETEC ” “ ETECB ” stand for “ BAY11-7082+DEFB118 ” “ ETECD ” stand for “ ETEC+DEFB118 ”

NF*-κ*B is the most critical transcription factor involved in various inflammatory signaling. So, we explore that if the DEFB118-modulated inflammatory response in IPEC-J2 cells were associated with the NF-κB signaling pathway. The results showed that both DEFB118 and BAY11-7082 significantly abolished the ETEC-induced inflammatory responses, indicated by decreases in cell apoptosis such as apoptosis rate, critical apoptosis-related genes (caspase 3, caspase 8, and caspase 9) and inflammatory cytokines such as the IL-1β, IL-6, and TNFα (Fig. 6). Moreover, both the DEFB118 and BAY11-7082 improved the abundance of ZO-1 in IPEC-J2 cells upon ETEC (Fig. 7A). Finally, we investigated the impacts of DEFB118 on the abundance of critical signaling proteins involved in the NF*-κ*B -induced inflammatory response. As shown in Fig. 7B, ETEC challenge acutely elevated the abundance of phosphorylated I*κ*B-α and NF*-κ*B; however, IPEC-J2 cells treated with DEFB118 and BAY11-7082 significantly decreased their phosphorylation.

## Discussion

Enterotoxigenic *Escherichia coli* (ETEC) is one of the main pathogens that cause post-weaning diarrhea (PWD) [33, 34]. Post-weaning diarrhea is an acute and highly contagious disease in piglets and characterized by watery diarrhea, dehydration and even death [35, 36], resulting in significant economic loss to the global pig industry [37]. Among the different ETEC, those expressing the F4+ fimbrial antigen are the most prevalent form of ETEC infection [38]. These fimbriate mediate the adhesion of ETEC to the host epithelial cells, enabling colonization of the small intestine [39]. Subsequently, heat-labile (LT) and heat-stable (STa/b) enterotoxins are secreted, which induce severe diarrhea [40]. In addition, ETEC derived endotoxins (such as the lipopolysaccharide) can stimulate the release of a variety of proinflammatory cytokines and other soluble factors, leading to systemic inflammation [41]. IPEC - J2 cell line has typical epithelial cell characteristics, which is a permitted host of symbiotic bacteria and intestinal pathogens, and is an excellent model for studying the interaction between bacteria and pig intestinal epithelial cells [42, 43]. Previous studies indicated that LT enhanced adherence of ETEC to IPEC-J2 cells [44, 45], STa may play a major role in ETEC-induced cell proliferation, cell apoptosis, destroyed cell barriers in IPEC-J2 cell [46, 47]. In addition, LT could increase expression level of pro-inflammatory cytokines (IL-8 and TNF-α) by activating NF-κB in HCT-8 cells [48], STb could induce intestinal barrier dysfunction in T84 cell [49–51], and induce apoptosis in intestinal epithelial cell lines (HRT-18 and IEC-18 cells) [52]. More studies are required to understand the effect of different virulence factors in pigs and other species. In the last decades, antibiotics have been widely used to treat the ETEC-induced diarrhea and inflammation in the swine industry. However, antibiotics, the most commonly applied control strategies, have been restricted in many countries due to the induction of antimicrobial resistance [53–55]. Therefore, novel avenues to prevent various bacteria-induced inflammation and intestinal epithelium disruption are urgently needed.

Previous studies have indicated that antimicrobial peptides (AMPs) are one of the most promising alternatives to antibiotics due to broad spectrum and a low propensity for developing resistance [56–66]. Moreover, AMPs can also act as an immunomodulator that plays a critical role in regulating the host innate immunity [67–69]. DEFB118 is a novel AMP, identified from epididymal epithelium and showed antibacterial activity against *E. coli* [31]. In the present study, we explored its protective effect on intestinal epithelial cells exposure to ETEC. We showed that DEFB118 can alleviate ETEC-induced inflammation in intestinal epithelial cells through inhibition of the NF-κB signaling pathway, resulting in reduced secretion of inflammatory cytokines and decreased cell apoptosis.

Mucosal epithelial barrier is the first line of defense against the invasion of intestinal pathogenic microorganisms and toxins [70]. As an important part of the intestinal mucosal barrier, changes of tight junction protein can cause abnormal intestinal barrier function and affect the intestinal health [71]. Pathogenic microorganisms (such as pathogenic Escherichia coli, epidemic diarrhea virus, etc.) can cause the expression of tight junction protein in intestinal epithelial cells decrease and increase the permeability [49, 50, 72, 73]. It is well known that the defective intestinal TJ barrier allows paracellular permeation of luminal antigens which can initiate or propagate the inflammatory responses [74]. ZO-1 (zonula occludens-1) is an important tight junction protein [75]. In this study, we found that DEFB118 pretreatment restored the abundance of ZO-1 in IPEC-J2 cells induced by ETEC. Therefore, the protective effect of DEFB118 on the intestinal barrier may be partly explained by the increased abundance of ZO-1 protein.

Apoptosis is known as programmed cell death (PCD). It is a kind of suicidal behavior under physiological and pathological conditions, which occurs after cell death process activated by various intracellular and extracellular signals [76]. Caspase is a cysteine protease family, which plays a key role in the process of apoptosis. Caspase-3 is the most critical protease downstream of the apoptosis cascade. It plays a central role in controlling apoptosis and plays a key role in activating the specific morphological and physiological changes of apoptosis [77]. Caspase-3 mediates apoptosis through exogenous activation pathway and endogenous activation pathway [78]. Exogenous pathway is mainly mediated by the death receptor; the endogenous activation pathway is mediated by mitochondria [79]. Caspase 3 is activated by Caspase 8 in the death receptor pathway and Caspase 9 in the mitochondrial pathway [80]. Previous studies have shown that ETEC can induce apoptosis [81–83]. The present study suggest that ETEC causes an increase in the expressions of caspase-3, caspase-8, and caspase-9. At the same time, the number of IPEC-J2 cells with early apoptosis, late apoptosis, and total apoptosis was also increased. However, these changes could be counteracted by DEFB118 pretreatments, suggesting the protective role of DEFB118 against intestinal barrier damage. This may be due to inhibition of caspase-3 gene expression by inhibiting caspase-8 gene expression in the death receptor pathway and caspase-9 gene expression in the mitochondrial pathway, thus inhibiting apoptosis induced by ETEC.

In order to further explore the anti-inflammatory mechanism of DEFB118, we used BAY11-7082 (an inhibitor of IκB-α phosphorylation and NF-κB) to investigate whether NF-κB signaling pathway is involved. Nuclear factor kappa B (NF-κB) is a multi- subunit nuclear transcription factor, which plays an important role in the regulation of many genes, including immune and inflammation, promoting or inhibiting the expression of chemotaxis and related apoptotic proteins, cell proliferation and tumorigenesis [84, 85]. When cells are in the resting state, NF-κB is inactive due to the existence of IκB-α. When cells are stimulated, IκB-α phosphorylates and rapidly degrades, activating NF-κB and transferring to nucleus. This translocation leads to the transcription and expression of inflammation-related genes [86, 87]. In agreement with previous studies [88, 89], elevated level of phosphorylated NF-κB protein expression was documented in the IPEC-J2 cells induced by ETEC. However, the pretrement of DEFB118 inhibited NF-κB phosphorylation. Additionally, it also inhibited IκB-α phosphorylation, which further downregulated NF-κB activity. BAY11-7082 was described as an irreversible inhibitor of the NF-κB pathway. It acts by inhibiting TNF-α-induced phosphorylation of IκB-α, resulting in decreased NF-κB and decreases expression of adhesion molecules [90]. And BAY11-7082 has been reported to display broad-spectrum anti-inflammatory activities and influence various physiological processes [91, 92]. In this study, we found that BAY11-7082 alleviate ETEC-induced inflammation in intestinal epithelial cells through inhibition of the NF-κB signaling pathway, which results in suppressing of inflammatory cytokines secretion and cell apoptosis. Interestingly, the effect of BAY11-7082 pretreatment was similar to that of DEFB118 pretreatment.

## Conclusion

DEFB118 can alleviate ETEC-induced inflammation in IPEC-J2 cells through inhibition of the NF-κB signaling pathway, which results in suppressing of inflammatory cytokines secretion and cell apoptosis. The beneficial effect of DEFB118 will help for rational section of novel anti-inflammatory agent for piglets

## Data Availability

The datasets generated during and analyzed during the current study are not publicly available due to internal regulations but are available from the corresponding author on reasonable request.

## Abbreviations

IPEC-J2: the porcine jejunum epithelial cell line
FBS: Fetal bovine serum
DEFB118: defensin beta 118
ETEC: enterotoxigenic *Escherichia coli*
IL-1β: interleukin-1β
IL-6: interleukin-6
TNF-α: tumor necrosis factor-α
ZO-1: tight-junction protein zonula occludens-1
NF-κB: nuclear factor-kappaB
IκB-α: factor-kappa-B inhibitor alpha

## References

[1] Liu Y, Chen F, Odle J, Lin X, Jacobi SK, Zhu H, et al. Fish oil enhances intestinal integrity and inhibits tlr4 and nod2 signaling pathways in weaned pigs after lps challenge. J Nutr. 2012. 10.3945/jn.112.164947. 10.3945/jn.112.164947 23014495

[2] UlluwishewaD ARC, McNabb WC, Moughan PJ, Wells JM, Roy NC. Regulation of tight junction permeability by intestinal bacteria and dietary components. J Nutr. 2011. 10.3945/jn.110.135657. 10.3945/jn.110.135657 21430248

[3] Segrist E, Cherry S. Using diverse model systems to define intestinal epithelial defenses to enteric viral infections. Cell Host Microbe. 2020. 10.1016/j.chom.2020.02.003. 10.1016/j.chom.2020.02.003 PMC7735531 32164844

[4] Manthey CF, Calabio CB, Wosinski A, Hanson EM, Vallance BA, Groisman A, et al. Indispensable functions of abl and pdgf receptor kinases in epithelial adherence of attaching/effacing pathogens under physiological conditions. Am J Physiol Cell Physiol. 2014. 10.1152/ajpcell.00013.2014. 10.1152/ajpcell.00013.2014 PMC4101622 24848114

[5] Petri WA, Miller M, Binder HJ, Levine MM, Dillingham R, Guerrant RL. Enteric infections, diarrhea, and their impact on function and development. J Clin Invest. 2008. 10.1172/JCI34005. 10.1172/JCI34005 PMC2276781 18382740

[6] Verhelst R, Schroyen M, Buys N, Niewold T. Dietary polyphenols reduce diarrhea in enterotoxigenic escherichia coli (ETEC) infected post-weaning piglets. Livest Sci. 2014. 10.1016/j.livsci.2013.11.026.

[7] Luise D, Lauridsen C, Bosi P, Trevisi P. Methodology and application of escherichia coli f4 and f18 encoding infection models in post-weaning pigs. J Animal Sci Biotechnol. 2019. 10.1186/s40104-019-0352-7. 10.1186/s40104-019-0352-7 PMC6567477 31210932

[8] Zhang W, Zhao M, Ruesch L, Omot A, Francis D. Prevalence of virulence genes in escherichia coli strains recently isolated from young pigs with diarrhea in the us. Vet Microbiol. 2007. 10.1016/j.vetmic.2007.02.018. 10.1016/j.vetmic.2007.02.018 17368762

[9] Svennerholm AM. From cholera to enterotoxigenic Escherichia coli (ETEC) vaccine development. Indian J Med Res. 2011. 10.1586/14760584.2014.905745. PMC3089050 21415493

[10] Gonzales L, Sanchez S, Zambrana S, Iñiguez V, Wiklund G, Svennerholm AM, et al. Molecular characterization of enterotoxigenic escherichia coli etec isolated from children with diarrhea during a four year period (2007-2010) in bolivia. J Clin Microbiol. 2013. 10.1128/JCM.02971-12. 10.1128/JCM.02971-12 PMC3666779 23390275

[11] Lin DM, Koskella B, Lin HC. Phage therapy: An alternative to antibiotics in the age of multi-drug resistance. World J Gastrointest Pharmacol Ther. 2017. 10.4292/wjgpt.v8.i3.16212. 10.4292/wjgpt.v8.i3.162 PMC5547374 28828194

[12] Aiyegoro O, Adewusi A, Oyedemi S, Akinpelu D, Okoh A. Interactions of antibiotics and methanolic crude extracts of Afzelia Africana (Smith.) against drug resistance bacterial isolates. Int J Mol Sci. 2011. 10.3390/ijms12074477. 10.3390/ijms12074477 PMC3155364 21845091

[13] Yu G, Baeder DY, Regoes RR, Rolff J. Predicting drug resistance evolution: insights from antimicrobial peptides and antibiotics. Proc Biol Sci. 2018. 10.1098/rspb.2017.2687. 10.1098/rspb.2017.2687 PMC5879628 29540517

[14] Tlaskalová-Hogenová H, Farré-Castany MA, Stĕpánková R, Kozáková H, Tucková L, Funda DP, et al. The gut as a lymphoepithelial organ: the role of intestinal epithelial cells in mucosal immunity. Folia Microbiol. 1995. 10.1007/BF02814746. 10.1007/BF02814746 8763152

[15] Vora P, Youdim P, Thomas LS, Fukata M, Abreu MT. β-Defensin-2 Expression Is Regulated by TLR Signaling in Intestinal Epithelial Cells. J Immunol. 2004. 10.4049/jimmunol.173.9.5398. 10.4049/jimmunol.173.9.5398 15494486

[16] Wehkamp J, Stange EF. A new look at Crohn's disease: breakdown of the mucosal antibacterial defense. Ann N Y Acad Sci. 2006. 10.1196/annals.1326.030. 10.1196/annals.1326.030 17057212

[17] Moeser AJ, Pohl CS, Rajput M. Weaning stress and gastrointestinal barrier development: implications for lifelong gut health in pigs. Animal Nutrition. 2017. 10.1016/j.aninu.2017.06.003. 10.1016/j.aninu.2017.06.003 PMC5941262 29767141

[18] Ganz T, Selsted ME, Szklarek D, Harwig SS, Daher K, Bainton DF, et al. Defensins. natural peptide antibiotics of human neutrophils. J Clin Investig. 1985. 10.1172/JCI112120. 10.1172/JCI112120 PMC424093 2997278

[19] Yang D, Biragyn A, Kwak LW, Oppenheim JJ. Mammalian defensins in immunity: more than just microbicidal. Trends Immunol. 2002. 10.1016/s1471-4906(02)02246-9. 10.1016/s1471-4906(02)02246-9 12072367

[20] Schutte BC, Mccray PB. β-defensins in lung host defense. Annu Rev Physiol. 2002. 10.1146/annurev.physiol.64.081501.134340. 10.1146/annurev.physiol.64.081501.134340 11826286

[21] Patil A, Hughes AL, Zhang G (2004). Rapid evolution and diversification of mammalian alpha-defensins as revealed by comparative analysis of rodent and primate genes. Physiol Genomics.

[22] Semple CA, Rolfe M, Dorin JR. Duplication and selection in the evolution of primate beta-defensin genes. Genome Biol. 2003. 10.1186/gb-2003-4-5-r31. 10.1186/gb-2003-4-5-r31 PMC156587 12734011

[23] Zhu S, Gao B. Evolutionary origin of β-defensins. Dev Comp Immunol. 2013. 10.1016/j.dci.2012.02.011. 10.1016/j.dci.2012.02.011 22369779

[24] Maiti S, Patro S, Purohit S, Jain S, Dey N. Effective control of Salmonella infections by employing combinations of recombinant antimicrobial human β-defensins HBD-1 and HBD-2. Antimicrob Agents Chemother. 2014. 10.1128/AAC.03628-14. 10.1128/AAC.03628-14 PMC4249419 25199778

[25] Ma D, Lin L, Zhang K, Han Z, Shao Y, X. Liu, et al. Three novel Anas platyrhynchos avian β-defensins, upregulated by duck hepatitis virus, with antibacterial and antiviral activities. Mol Immunol 2011; doi: 10.1016/j.molimm.2011.07.019 10.1016/j.molimm.2011.07.019 21856003

[26] Kolar SS, Baidouri H, Hanlon S, Mcdermott AM. Protective role of murine β-defensins 3 and 4 and cathelin-related antimicrobial peptide in Fusarium solani keratitis. Infect Immun. 2013. 10.1128/IAI.00179-13. 10.1128/IAI.00179-13 PMC3719577 23670560

[27] Xie KH, Su GQ, Chen DW, Yu B, Mao XB, Huang ZQ, et al. β-Defensin 129 Attenuates Bacterial Endotoxin-Induced Inflammation and Intestinal Epithelial Cell Apoptosis. Front Immunol. 2019. 10.3389/fimmu.2019.02333. 10.3389/fimmu.2019.02333 PMC6787771 31636641

[28] Yu H, Dong J, Gu Y, Liu H, Xin A, Shi H, et al. The novel human β-defensin 114 regulates lipopolysaccharide (LPS)-mediated inflammation and protects sperm from motility loss. J Biol Chem. 2013. 10.1074/jbc.M112.411884. 10.1074/jbc.M112.411884 PMC3636911 23482568

[29] Qiang L, Hamil KG, Sivashanmugam P, Grossman G, Soundararajan R, Rao AJ, et al. Primate epididymis-specific proteins: characterization of ESC42, a novel protein containing a trefoil-like motif in monkey and human. Endocrinology. 2001. 10.1210/endo.142.10.8422. 10.1210/endo.142.10.8422 11564719

[30] Yenugu S, Hamil KG, Radhakrishnan Y, French FS, Hall SH. The androgen-regulated epididymal sperm-binding protein, human beta-defensin 118 (DEFB118) (formerly ESC42), is an antimicrobial beta-defensin. Endocrinology. 2004. 10.1210/en.2003-1698. 10.1210/en.2003-1698 15033915

[31] Lin Q, Xie KH, Chen DW, Yu B, Mao X, Huang Z, et al. Expression and Functional Characterization of a Novel Antimicrobial Peptide: Human Beta-Defensin 118. Biomed Res Int. 2020. 10.1155/2020/1395304. 10.1155/2020/1395304 PMC7673234 33224970

[32] Fleige S, Walf V, Huch S, Prgomet C, Pfaffl MW. Comparison of relative mrna quantification models and the impact of rna integrity in quantitative real-time RT-PCR. Biotechnol Lett. 2006. 10.1007/s10529-006-9127-. 10.1007/s10529-006-9127-2 16900335

[33] Nagy B, Fekete PZ. Enterotoxigenic Escherichia coli (ETEC) in farm animals. Vet Res. 1999. 10.1111/j.1740-8261.1999.tb01909.x. 10367358

[34] Fairbrother JM, Nadeau E, Gyles CL. Escherichia coli in postweaning diarrhea in pigs: an update on bacterial types, pathogenesis, and prevention strategies. Anim Health Res Rev. 2005. 10.1079/ahr2005105.34. 10.1079/ahr2005105 16164007

[35] Svensmark B, Nielsen K, Willeberg P, Jorsal SE (1989). Epidemiological studies of piglet diarrhoea in intensively managed danish sow herds. ii. post-weaning diarrhoea. Acta Vet Scand.

[36] Lyutskanov M (2011). Epidemiological characteristics of post-weaning diarrhoea associated with toxin-producing escherichia coli in large intensive pig farms. Trakia J Sci.

[37] Liu M, Zhang Y, Zhang D, Bai Y, Li Y. Immunogenicity and protective efficacy of enterotoxigenic escherichia coli (ETEC) total rna against etec challenge in a mouse model. Sci Rep. 2020. 10.1038/s41598-020-77551-8. 10.1038/s41598-020-77551-8 PMC7689534 33239756

[38] Rapacz J, Hasler-Rapacz J. Polymorphism and inheritance of swine small intestinal receptors mediating adhesion of three serological variants of Escherichia coli-producing K88 pilus antigen. Anim Genet. 1986. 10.1111/j.1365-2052.1986.tb00724.x40. 10.1111/j.1365-2052.1986.tb00724.x 2881506

[39] Vanden Broeck W, Cox E, Goddeeris BM. Receptor-specific binding of purified f4 to isolated villi. Vet Microbiol. 1999. 10.1016/s0378-1135(99)00076-0. 10.1016/s0378-1135(99)00076-0 10510044

[40] Sun Y, Kim SW. Intestinal challenge with enterotoxigenic Escherichia coli in pigs, and nutritional intervention to prevent postweaning diarrhea. Animal Nutrition. 2017. 10.1016/j.aninu.2017.10.00142. 10.1016/j.aninu.2017.10.001 PMC5941267 29767133

[41] Lien E, Means TK, Heine H, Yoshimura A, Kusumoto S, Fukase K, et al. Toll-like receptor 4 imparts ligand-specific recognition of bacterial lipopolysaccharide. J Clin Invest. 2000. 10.1172/JCI8541. 10.1172/JCI8541 PMC289161 10683379

[42] Schierack P, Nordhoff M, Pollmann M, Weyrauch KD, Amasheh S, Lodemann U, et al. Characterization of a porcine intestinal epithelial cell line for in vitro studies of microbial pathogenesis in swine. Histochem Cell Biol. 2006. 10.1007/s00418-005-0067-z. 10.1007/s00418-005-0067-z 16215741

[43] Zakrzewski SS, Richter JF, Krug SM, Jebautzke B, Lee IF, Rieger et al. Improved cell line IPEC-J2, characterized as a model for porcine jejunal epithelium. Plos One 2013; doi: 10.1371/journal.pone.0079643 10.1371/journal.pone.0079643 PMC3829867 24260272

[44] Fekete PZ, Mateo KS, Zhang W, Moxley RA, Kaushik RS, Francis DH. Both enzymatic and non-enzymatic properties of heat-labile enterotoxin are responsible for LT-enhanced adherence of enterotoxigenic Escherichia coli to porcine IPEC-J2 cells. Vet Microbiol. 2013. 10.1016/j.vetmic.2013.02.019. 10.1016/j.vetmic.2013.02.019 23517763

[45] Wijemanne P, Moxley RA. Glucose significantly enhances enterotoxigenic Escherichia coli adherence to intestinal epithelial cells through its effects on heat-labile enterotoxin production. PLoS One. 2014. 10.1371/journal.pone.0113230. 10.1371/journal.pone.0113230 PMC4237375 25409235

[46] Zhou JY, Huang DG, Gao CQ, Yan HC, Zou SG, Wang XQ. Heat-stable enterotoxin inhibits intestinal stem cell expansion to disrupt the intestinal integrity by downregulating the Wnt/β-catenin pathway. Stem Cells. 2021. 10.1002/stem.3324. 10.1002/stem.3324 33373490

[47] Zhu J, Yin X, Yu H, Zhao L, Sabour P, Gong J. Involvement of quorum sensing and heat-stable enterotoxin a in cell damage caused by a porcine enterotoxigenic Escherichia coli strain. Infect Immun. 2011. 10.1128/IAI.01281-10. 10.1128/IAI.01281-10 PMC3067552 21300771

[48] Wang X, Gao X, Hardwidge PR. Heat-labile enterotoxin-induced activation of NF-κB and MAPK pathways in intestinal epithelial cells impacts enterotoxigenic Escherichia coli (ETEC) adherence. Cell Microbiol. 2012. 10.1111/j.1462-5822.2012.01793.x. 10.1111/j.1462-5822.2012.01793.x PMC3391543 22452361

[49] Nassour H, Dubreuil JD. Escherichia coli STb enterotoxin dislodges claudin-1 from epithelial tight junctions. PLoS One. 2014. 10.1371/journal.pone.0113273. 10.1371/journal.pone.0113273 PMC4237405 25409315

[50] Ngendahayo Mukiza C, Dubreuil JD. Escherichia coli heat-stable toxin b impairs intestinal epithelial barrier function by altering tight junction proteins. Infect Immun. 2013. 10.1128/IAI.00455-13. 10.1128/IAI.00455-13 PMC3719558 23716609

[51] Nakashima R, Kamata Y, Nishikawa Y. Effects of Escherichia coli heat-stable enterotoxin and guanylin on the barrier integrity of intestinal epithelial T84 cells. Vet Immunol Immunopathol. 2013. 10.1016/j.vetimm.2012.09.026. 10.1016/j.vetimm.2012.09.026 23078906

[52] Syed HC, Dubreuil JD. Escherichia coli STb toxin induces apoptosis in intestinal epithelial cell lines. Microb Pathog. 2012. 10.1016/j.micpath.2012.06.003. 10.1016/j.micpath.2012.06.003 22771838

[53] Luppi A, Bonilauri P, Dottori M, Gherpelli Y, Biasi G, Merialdi G, et al. Antimicrobial resistance of F4+ Escherichia coli isolated from Swine in Italy. Transbound Emerg Dis. 2015. 10.1111/tbed.12081. 10.1111/tbed.12081 23906344

[54] Rhouma M, Fairbrother JM, Beaudry F, Letellier A. Post weaning diarrhea in pigs: risk factors and non-colistin-based control strategies. Acta Vet Scand. 2017. 10.1186/s13028-017-0299-7. 10.1186/s13028-017-0299-7 PMC5437690 28526080

[55] Jahanbakhsh S, Smith MG, Kohan-Ghadr HR, Letellier A, Abraham S, Trott DJ, et al. Dynamics of extended-spectrum cephalosporin resistance in pathogenic escherichia coli isolated from diseased pigs in quebec, canada. Int J Antimicrob Agents. 2016. 10.1016/j.ijantimicag.2016.05.001. 10.1016/j.ijantimicag.2016.05.001 27286922

[56] Lin Q, Su GQ, Wu AM, Chen DW, Yu B, Mao XB, et al. Bombyx mori gloverin a2 alleviates enterotoxigenic escherichia coli-induced inflammation and intestinal mucosa disruption. Antimicrob Resist Infect Control. 2019. 10.1186/s13756-019-0651-y. 10.1186/s13756-019-0651-y PMC6878672 31788236

[57] Su GQ, Xie KH, Chen DW, Yu B, Mao XB, Huang ZQ, et al. Differential expression, molecular cloning, and characterization of porcine beta defensin 114. J Animal Sci Biotechnol. 2019. 10.1186/s40104-019-0367-0. 10.1186/s40104-019-0367-0 PMC6639935 31360462

[58] Semple F, Webb S, Li HN, Patel HB, Perretti M, Jackson IJ, et al. Human β-defensin 3 has immunosuppressive activity in vitro and in vivo. Eur J Immunol. 2010. 10.1002/eji.200940041. 10.1002/eji.200940041 PMC2948537 20104491

[59] Cao L, Dai C, Li Z, Fan Z, Song Y, Wu Y, et al. Antibacterial activity and mechanism of a scorpion venom peptide derivative in vitro and in vivo. Plos One. 2012. 10.1371/journal.pone.0040135. 10.1371/journal.pone.0040135 PMC3390344 22792229

[60] Radek K, Gallo R. Antimicrobial peptides: natural effectors of the innate immune system. Semin Immunopathol. 2007. 10.1007/s00281-007-0064-5. 10.1007/s00281-007-0064-5 17621952

[61] Nguyen LT, Haney EF, Vogel HJ. The expanding scope of antimicrobial peptide structures and their modes of action. Trends Biotechnol. 2011. 10.1016/j.tibtech.2011.05.001. 10.1016/j.tibtech.2011.05.001 21680034

[62] Teixeira V, Feio MJ, Bastos M. Role of lipids in the interaction of anti-microbial peptides with membranes. Prog Lipid Res. 2012. 10.1016/j.plipres.2011.12.005. 10.1016/j.plipres.2011.12.005 22245454

[63] Huang Y, Huang J, Chen Y. Alpha-helical cationic antimicrobial peptides: relationships of structure and function. Protein Cell. 2010. 10.1007/s13238-010-0004-3. 10.1007/s13238-010-0004-3 PMC4875170 21203984

[64] Shai Y, Oren Z. From "carpet" mechanism to de-novo designed diastereo-meric cell-selective antimicrobial peptides. Peptides. 2001. 10.1016/s0196-9781(01)00498-3. 10.1016/s0196-9781(01)00498-3 11587791

[65] Rotem S, Mor M. Antimicrobial peptide mimics for improved therapeutic properties. BBA-Biomembranes. 2008. 10.1016/j.bbamem. 10.1016/j.bbamem.2008.10.020 19028449

[66] Hancock RE. The bacterial outer membrane as a drug barrier. Trends Microbiol. 1997. 10.1016/S0966-842X(97)81773-8. 10.1016/S0966-842X(97)81773-8 9025234

[67] Beisswenger C, Bals R. Functions of antimicrobial peptides in host defense and immunity. Curr Protein Pept Sci. 2005. 10.2174/1389203054065428. 10.2174/1389203054065428 15974951

[68] Prasad BD, Sahni S, Ranjan T, Kumari D. Antimicrobial proteins: key components of innate immunity. Curr J Appl Sci Technol. 2019. 10.9734/cjast/2019/v36i330236.

[69] Bergman P, Raqib R, Rekha RS, Agerberth B, Gudmundsson GH. Host directed therapy against infection by boosting innate immunity. Front Immunol. 2020. 10.3389/fimmu.2020.01209. 10.3389/fimmu.2020.01209 PMC7304486 32595649

[70] Pitman RS, Blumberg RS. First line of defense: the role of the intestinal epithelium as an active component of the mucosal immune system. J Gastroenterol. 2000. 10.1007/s005350070017. 10.1007/s005350070017 11085489

[71] Xu CM, Li XM, Qin BZ, Liu B. Effect of tight junction protein of intestinal epithelium and permeability of colonic mucosa in pathogenesis of injured colonic barrier during chronic recovery stage of rats with inflammatory bowel disease. Asian Pac J Trop Med. 2016. 10.1016/j.apjtm.2016.01.001. 10.1016/j.apjtm.2016.01.001 26919945

[72] Campbell HK, Maiers JL, Demali KA. Interplay between tight junctions & adherens junctions. Exp Cell Res. 2017. 10.1016/j.yexcr.2017.03.061. 10.1016/j.yexcr.2017.03.061 PMC5544570 28372972

[73] Salim SY, Sderholm JD. Importance of disrupted intestinal barrier in in-flammatory bowel diseases. Inflamm Bowel Dis. 2011. 10.1002/ibd.21403. 10.1002/ibd.21403 20725949

[74] Hollander D. Intestinal permeability, leaky gut, and intestinal disorders. Curr Gastroenterol Rep. 1999. 10.1007/s11894-999-0023-5. 10.1007/s11894-999-0023-5 10980980

[75] Zong Q, Qin W, Huo Y, Wu S, Bao W (2018). Progress in tight junction protein of pig intestine. Zhejiang Agric J.

[76] Miu L, Yao L. Research progress of mitochondrial related apoptosis proteins. Basic medicine and clinical. 2012;32:837-40. 10.16352/j.issn.1001-6325.2012.07.012.

[77] Wu Y, Zheng W, Li Y. The role of Caspase-3 in vascular endothelial cell apoptosis. Southwest Military Doctor. 2013. 10.3969/j.issn.1672-7193.2013.04.017.

[78] Meggiato T, Calabrese F, De Cesare CM, Baliello E, Valente M, Del Favero G. C-JUN and CPP32 (CASPASE 3) in human pancreatic cancer: relation to cell proliferation and death. Pancreas. 2003. 10.1097/00006676-200301000-00011. 10.1097/00006676-200301000-00011 12499919

[79] Khan KH, Blanco-Codesido M, Molife LR. Cancer therapeutics: targeting the apoptotic pathway. Critical Rev Oncol. 2014. 10.1016/j.critrevonc.2013.12.012. 10.1016/j.critrevonc.2013.12.012 24507955

[80] Dai X, Li Z, Linhua J. Progress in caspase, a protein associated with apoptosis. Chin J Modern Med. 2010. 10.3969/j.issn.1672-9463.2010.04.064.

[81] Xia Y, Chen S, Zhao Y, Chen S, Huang R, Zhu G, et al. GABA attenuates ETEC-induced intestinal epithelial cell apoptosis involving gabaar signaling and the ampk-autophagy pathway. Food Funct. 2019. 10.1039/c9fo01863h. 10.1039/c9fo01863h 31670355

[82] Xia Y, Bin Y, Liu S, Chen S, Yin J, Liu G. Enterotoxigenic escherichia coli infection promotes apoptosis in piglets. Microb Pathog. 2018. 10.1016/j.micpath.2018.09.032. 10.1016/j.micpath.2018.09.032 30243552

[83] Li Y, Wang J, Li Y, Wu H, Zhao S, Yu Q. Protecting intestinal epithelial cells against deoxynivalenol and e. coli damage by recombinant porcine IL-22. Vet Microbiol. 2019. 10.1016/j.vetmic.2019.02.027. 10.1016/j.vetmic.2019.02.027 PMC7172643 30955803

[84] Soonmi W, Iqbal S, Peterson BL, Bushra W, Kahn JS, Stein DG. Vitamin D prevents hypoxia/reoxygenation-induced blood-brain barrier disruption via vitamin D receptor-mediated NF-kB signaling pathways. PLoS One. 2015. 10.1371/journal.pone.0122821. 10.1371/journal.pone.0122821 PMC4376709 25815722

[85] Lee JW, Bae CJ, Choi YJ, Kim KI, Kim NH, Lee HJ, et al. 3,4,5-Trihydroxycinnamic Acid Inhibits LPS-Induced iNOS Expression by Suppressing NF-κB Activation in BV2 Microglial Cells. Kor J Physiol Pharmacol. 2012. 10.4196/kjpp.2012.16.2.107. 10.4196/kjpp.2012.16.2.107 PMC3339285 22563255

[86] Lawrence T. The nuclear factor NF-kappaB pathway in inflammation. Cold Spring Harb Perspect Biol. 2009. 10.1101/cshperspect.a001651. 10.1101/cshperspect.a001651 PMC2882124 20457564

[87] Hsuan CF, Hsu HF, Tseng WK, Lee TL, Wei YF, Hsu KL, et al. Glossogyne tenuifolia Extract Inhibits TNF-α-Induced Expression of Adhesion Molecules in Human Umbilical Vein Endothelial Cells via Blocking the NF-kB Signaling Pathway. Molecules. 2015. 10.3390/molecules200916908. 10.3390/molecules200916908 PMC6332270 26393541

[88] Shi Y, Li W, Han L, Gao Y, Wang H, Jin F. Protective effect of antimicrobial peptide LL37 on lipopolysaccharide induced inflammatory injury of rat alveolar macrophages. Int J Respiratory. 2019. 10.3760/cma.j.issn.1673-436X.2019.12.002.

[89] Roselli M, Finamore A, Hynönen U, Palva A, Mengheri E. Differential protection by cell wall components of Lactobacillus amylovorus DSM 16698^T^ against alterations of membrane barrier and NF-kB activation induced by enterotoxigenic F4^+^ Escherichia coli on intestinal cells. BMC Microbiol. 2016. 10.1186/s12866-016-0847-8. 10.1186/s12866-016-0847-8 PMC5041403 27688074

[90] Pierce JW, Schoenleber R, Jesmok G, Best J, Moore SA, Collins T, et al. Novel inhibitors of cytokine-induced IkappaBalpha phosphorylation and endothelial cell adhesion molecule expression show anti-inflammatory effects in vivo. J Biol Chem. 1997. 10.1074/jbc.272.34.21096. 10.1074/jbc.272.34.21096 9261113

[91] Lee J, Rhee MH, Kim E, Cho JY. BAY 11-7082 is a broad-spectrum inhibitor with anti-inflammatory activity against multiple targets. Mediators Inflamm. 2012. 10.1155/2012/416036. 10.1155/2012/416036 PMC3382285 22745523

[92] Xu C, Zhang Y, Sutrisno L, Yang L, Chen R, Sung KL. Bay11-7082 facilitates wound healing by antagonizing mechanical injury- and TNF-α-induced expression of MMPs in posterior cruciate ligament. Connect Tissue Res. 2018. 10.1080/03008207.2018.1512978. 10.1080/03008207.2018.1512978 30372627

